# Community coverage of an antimalarial combination of artesunate and amodiaquine in Makamba Province, Burundi, nine months after its introduction

**DOI:** 10.1186/1475-2875-6-94

**Published:** 2007-07-18

**Authors:** Sibylle Gerstl, Sandra Cohuet, Kodjo Edoh, Christopher Brasher, Alexandre Lesage, Jean-Paul Guthmann, Francesco Checchi

**Affiliations:** 1 Epicentre, Paris, France; 2 Médecins sans Frontières-France, Paris, France; 3 Department of Infectious and Tropical Diseases, London School of Hygiene and Tropical Medicine, London, UK

## Abstract

**Background:**

In 2003, artesunate-amodiaquine (AS+AQ) was introduced as the new first-line treatment for uncomplicated malaria in Burundi. After confirmed diagnosis, treatment was delivered at subsidized prices in public health centres. Nine months after its implementation a study was carried out to assess whether children below five years of age with uncomplicated malaria were actually receiving AS+AQ.

**Methods:**

A community-based study was conducted in Makamba province. Randomly selected households containing one or more children under five with reported fever onset within fourteen days before the study date were eligible. Case-management information was collected based on caregiver recall. A case definition of symptomatic malaria from observations of children presenting a confirmed malaria episode on the day of the survey was developed. Based on this definition, those children who had probable malaria among those with fever onset in the 14 days prior to the study were identified retrospectively. Treatment coverage with AS+AQ was then estimated among these probable malaria cases.

**Results:**

Out of 195 children with fever on the day of the study, 92 were confirmed as true malaria cases and 103 tested negative. The combination of 'loss of appetite', 'sweating', 'shivering' and 'intermittent fever' yielded the highest possible positive predictive value, and was chosen as the case definition of malaria. Out of 526 children who had had fever 14 days prior to the survey, 165 (31.4%) were defined as probable malaria cases using this definition. Among them, 20 (14.1%) had been treated with AS+AQ, 10 with quinine (5%), 68 (41%) received non-malaria treatments, and 67 got traditional treatment or nothing (39.9%). Most people sought treatment from public health centres (23/99) followed by private clinics (15/99, 14.1%). The median price paid for AS+AQ was 0.5 US$.

**Conclusion:**

AS+AQ was the most common treatment for patients with probable malaria at public health centres, but coverage was low due to low health centre utilisation and apparently inappropriate prescribing. In addition, AS+AQ was given to patients at a price ten times higher than the subsidized price. The availability and proper use of ACTs should be monitored and maximized after their introduction in order to have a significant impact on the burden of malaria.

## Background

Artemisinin-based combination therapies (ACT) are recommended by WHO throughout sub-Saharan Africa as replacement for failing first-line antimalarials. Their advantages and potential to reduce malaria morbidity and mortality have been well described [1,2]. Substantial reductions of the burden of malaria have been partly attributed to ACT introduction in Thailand, Vietnam and South Africa, where these combinations have been in use for some years [3-5]. However, the benefit of these advantages hinges upon important requirements related to access to and use of these treatments.

In Burundi, malaria is the major health problem in terms of both morbidity and mortality [6]. During the last decade caseloads increased from 550,000 in 1991 to 2,8 million in 2001 in a population of 7 million [6,7]. Moreover, at the beginning of 2001 Burundi experienced a devastating *Plasmodium falciparum *epidemic with 630 to 1,190 deaths per day [8]. Efficacy studies carried out at the same time illustrated very high resistance levels to the commonly used first and second-line antimalarials [8]. As a consequence, in November 2003 Burundi introduced an artesunate-amodiaquine combination (AS+AQ), as the new first-line treatment for uncomplicated malaria.

In Makamba province, situated in the South of the country and one of the most affected by malaria, numerous agencies, including Médecins Sans Frontières (MSF), were involved in the financing, provision and monitoring of this new treatment. A full course of treatment, including both a confirmed diagnosis by means of a rapid test (Paracheck^®^) or a thick smear, and outpatient consultation fees, was theoretically available in public health centres at a heavily subsidized price (0.05 US$ for children below 5 years and 0.1 US$ for adults). In Burundi in 2004, the gross national income per capita was estimated at 90 US$ [9].

The deployment of AS+AQ as a new first-line antimalarial treatment in Burundi is an important first step in reducing malaria morbidity and mortality. However, this cannot occur unless a high proportion of cases receives prompt and adequate treatment with ACT (referred here as treatment coverage). Disappointing coverage may result from (i) low access to health care in the population affected and (ii) inadequate diagnosis, prescription and use of the new treatment in health centres. The Roll Back Malaria Initiative has set a goal of achieving 60% coverage of childhood fevers with effective, prompt antimalarial treatment. Nine months after its introduction in Burundi, a study was carried out to assess the coverage of AS+AQ among uncomplicated malaria cases in children less than five years of age.

## Methods

### Study setting

The study was carried out in Makamba province in the south of Burundi bordering Tanzania and lake Tanganyika (277,000 inhabitants) [10]. The province was affected by protracted armed conflict until 2002 when a ceasefire was signed. The basic administrative unit is the 'colline', equivalent to a village. Eighteen public health centres are operational. Malaria is the leading reported cause of morbidity, but different transmission strata are identified: the lake-side hyper-endemic area (< 1400 m), the plateau area at epidemic risk (1400–1800 m) and the mountain area with sporadic malaria cases (>1800 m). *Plasmodium falciparum *is the most common species observed. The Burundian Ministry of Health and local authorities approved the study.

### Study design

Classically, studies of antimalarial treatment coverage have used cross-sectional designs in which patients or caregivers of children are asked to report on past febrile episodes and on their management [11,12]. One advantage of this study design is that it requires a limited amount of time for data collection. However, caregiver reports can be poorly predictive of true childhood fever [13,14]. Furthermore, in Burundi malaria treatment is not administered to all fevers, but rather only to parasite-positive children fulfilling certain clinical criteria. In the absence of a validated biological test to identify such children retrospectively, an attempt was made to develop a symptom-based definition of probable uncomplicated malaria, that, though based merely on caregivers' verbal recall, would nonetheless reflect the true denominator of the coverage proportion, namely children with a true episode of uncomplicated malaria who would thus have been eligible to receive AS+AQ in public health centres.

Accordingly, two simultaneous community-based studies were carried out:

**(i) a cross sectional malaria case management study**. Here, households of children under five years with reported fever onset in the 14 days prior to the study day were sampled, and their caregivers were asked to recall the children's symptoms and case management.

**(ii) a study to develop a local, symptom-based retrospective case definition of malaria**. Children under five years reported as febrile on the day of the study were sampled. Among these, a gold standard diagnosis of true uncomplicated malaria was established, and caregivers were asked to describe the children's symptoms. Combining those symptoms most predictive of true malaria, the best possible symptom-based definition of malaria was identified, which was then applied to children in the case management sample so as to restrict the analysis to 'probable malaria cases'.

In practice, the two studies used partially overlapping samples, since many children with fever in the previous 14 days were also febrile on the day of the study.

### Cross-sectional study of malaria case-management

Households containing one or more children under five years of age, with reported fever onset one to fourteen days before the study date were eligible. Data were collected on all such children present in the household, so as to classify the level of access to health care in different areas within Makamba province (data not shown in this paper). Lot Quality Assurance Sampling rules were used to select an equal-sized and thus non-self-weighting sample of 24 households within each of the 18 health centre catchment areas ('lots') (SampleLQ v1.10) [15]. Within each lot, villages were allocated a number of households proportionately to their population size. Within each village, each household (defined as a group of people living together and eating from the same pot) was selected by picking a random direction at the centre of the village, walking to its edge while enumerating households along the way, and choosing one of these using a random number. Ineligible households were replaced with those nearest.

After providing written informed consent, caregivers were interviewed in Kirundi using a standardized, pre-piloted, back-translated questionnaire. Information was collected on household socio-demographics, signs and symptoms in the febrile children, and case management during febrile episodes.

Each observation was then aggregated into an overall province-wide sample, applying population weights so as to account for differing lot population sizes. The sample would thus consist of at least 432 children with a history of fever (18 lots × 24 households × at least one child per household). Even if only 1/3 of these had subsequently fitted the symptom-based malaria definition, this sample is still sufficient to estimate coverage with a precision no worse than ± 10%.

### Development of a retrospective symptom-based malaria case definition

While sampling households as part of the case-management study, any children with reported fever on the study date were identified. After obtaining written informed consent, caregivers were asked whether the children exhibited any of a pre-determined list of signs and symptoms. The same pre-determined list of signs and symptoms was used as for the case management study. In addition, clinical assistants examined the children for signs of malaria (e.g. splenomegaly, hepatomegaly, anaemia) and other febrile pathologies (e.g. respiratory infections, acute watery or bloody diarrhoea, abscesses, etc.), and thick blood slides were taken. An expert technician read all blood slides, and 5% were blindly re-read for quality control. For ethical reasons, sick children were treated on site or given a voucher for free treatment at the nearest health centre.

Febrile children with positive slides with parasite count ≥2,000/μL and no signs of a severe illness not due to malaria, (e.g. acute lower respiratory infection, acute or watery bloody diarrhoea) and children with positive slides with parasite count < 2,000/μL and no signs of another non-malaria febrile illness, were considered true malaria cases; all others were considered true negatives. Against this gold standard classification, various combinations of caregiver-reported symptoms (i.e. more than one symptom occurring together in the same child) were tested by calculating their positive (PPV) and negative (NPV) predictive values. In order to establish the most specific symptom-based definition of malaria PPV was favoured over NPV as the main function of this definition was to exclude as many false positive malaria cases as possible. The chosen combination of symptoms was then applied to the case management sample so as to retrospectively identify children whose reported fever was probably due to malaria.

Based on a Kenyan study [11], it was expected that about one third of children with reported fever would be febrile on the day of the survey, and thus that PPV and NPV would have been calculated on a sample of at least 144 cases.

### Data entry and analysis

Data were entered on EpiData version 3.0 (The EpiData Association, Odense, Denmark) and checked for inconsistencies. Analysis was done using Stata 8.0 (Stata Corporation, College Station, Texas, USA). The treatment coverage with AS+AQ among probable malaria cases and the case management of probable malaria cases were expressed as percentages weighted by population size. Probable malaria cases were taken as the denominator of the coverage estimate. A strict definition of coverage was used: AS+AQ prescription in a public health centre and by the second day after onset of fever (approximately 48 hours). A more lenient definition was also applied, namely having received AS+AQ under any circumstances.

### Ethical approval

The study was carried out with the permission of the Ministry of Health, Bujumbura Burundi. A written informed consent was obtained from all who participated in the study.

## Results

### Study profile

**Figure 1:**
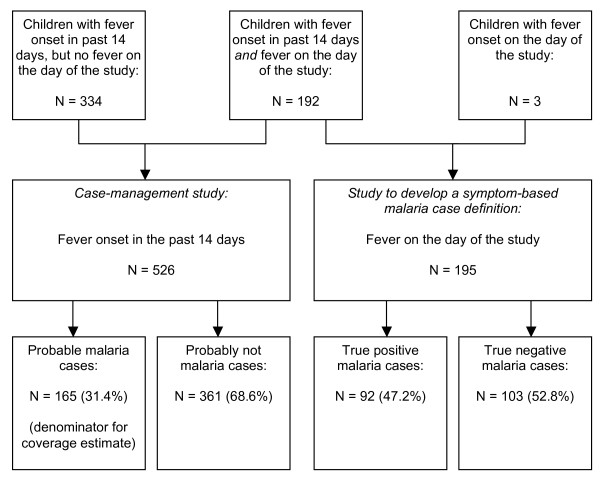
Study profile, Makamba province, Burundi, 2004.

Data collection took place between July 30 and August 20, 2004. A total of 526 children were included in the case management sample and 195 in the symptom-based malaria definition sample (Figure 1). Of the children in the malaria definition sample, 192/195 (98.5%) had fever onset in the previous 14 days, and were thus also part of the case-management sample. The two samples did not differ in baseline characteristics. The median age of the children was in both samples 2.3 years (p = 0.99) and the male to female sex ratio 0.96 and 0.88 respectively (p = 0.82). Children only included in the case-management sample were also similar to children in the malaria definition sample. Almost half of the caregivers were illiterate (44.9%, 236/526) and none had educational degrees beyond secondary school. Eighty-one percent (426/526) were subsistence farmers. Most were aware that malaria is transmitted by mosquitoes (86.9%, 457/526), but only 22.8% had a mosquito net at home (120/526).

### Development of a retrospective symptom-based malaria case definition

Out of the 195 children with fever on the study date, 92 were classified as true malaria cases (proportionate malarial morbidity 47.2%, 95%CI 38.5–55.9) and 103 as true negatives. Among all symptoms reported by the caregivers, loss of appetite, sweating, somnolence, shivering and intermittent fever had a PPV higher than 50% (Table 1). Abscess/ulcer also had a high PPV, but only occurred in 6/195 children and was excluded as clearly unrelated to malaria. The combination of 'loss of appetite' AND 'sweating' AND 'shivering' AND 'intermittent fever' yielded a relatively high PPV (62.5%), with a NPV of 55.6% and retrospectively classified 165/526 (31.4%) children in the case management sample as probable malaria cases (Figure 1, Table 1). This was thus chosen as the symptom-based definition of malaria. All other symptom combinations achieved lower PPV and classified a similar proportion of children as malaria cases, with the exception of 'loss of appetite' AND 'sweating' AND 'somnolence', which gave a PPV of 66.7% but only classified 56/526 (10.6%) children as malaria cases, and was not selected on this basis.

**Table 1:** Sensitivity, specificity and predictive values (positive-PPV and negative-NPV) of caregiver-reported signs and symptoms among children with reported fever on the study date, based on 92 true positive and 103 true negative malaria cases, Makamba province, Burundi, 2004.

Sign/Symptom	True positive malaria cases featuring this sign/symptom [n/92]	True negative malaria cases featuring this sign/symptom [n/103]	Sensitivity [%]	Specificity [%]	NPV [%]	PPV [%]
Abscess/ulcer*	5	1	6.6	97.7	50.3	75.0
Loss of appetite	55	40	59.8	55.1	57.0	57.9
Sweating	61	54	67.8	54.7	54.7	53.0
Somnolence	18	17	19.8	80.9	49.7	51.4
Shivering	52	50	56.5	49.4	49.4	51.0
Intermittent fever	90	87	97.8	2.3	50.0	50.9
Pallor	12	12	13.0	86.4	48.7	50.0
Rash*	8	8	8.7	91.0	49.1	50.0
Night cough	52	60	56.5	32.6	42.0	46.4
Convulsions	5	6	5.4	93.2	48.5	45.5
Watery diarrhoea	29	38	31.5	57.3	44.7	43.3
Vomiting	24	38	26.1	57.3	42.9	38.7
Jaundice	5	8	5.4	91.0	48.2	38.5
Headache	6	10	7.4	87.3	47.9	38.0
Bloody diarrhoea*	1	2	1.1	97.8	48.9	33.3
Loss of appetite AND sweating AND shivering AND intermittent fever	40	24	43.5	73.0	55.6	62.5

* symptoms excluded as they are related to another pathology

### Treatment coverage with AS+AQ among probable malaria cases

Among the 165 probable malaria cases, 40% were treated with traditional medicine or did not get any treatment, while 41% received drugs other than antimalarials (Table 2). Only 13 (9.4%, 95%CI 3.8–15.0) met the strict definition of coverage with AS+AQ, while 20 (14.1%, 95%CI 7.6–20.3) met the lenient definition. A further 10 received quinine (5.0%, 95%CI 1.7–8.4), the recommended second-line regimen in Burundi. Assuming these were justified prescriptions for suspected relapses, the antimalarial treatment coverage with either AS+AQ or quinine was 30/165 (19.1%, 95%CI 12.2–16.0). Among all children with a history of fever in the past 14 days, irrespective of whether they fitted the symptomatic malaria definition, 44/526 (7.9%, 95%CI 5.3–10.6) received AS+AQ. Coverage did not differ significantly among children who were only included in the case-management sample (n = 334; data not shown).

**Table 2:** Treatment coverage in probable malaria cases, Makamba province, Burundi, 2004 (n = 165)

Treatment	N	[%]*	95%CI
*Strict definition of AS+AQ coverage*^†^			
AS+AQ	13	9.4	3.8–15.0
Quinine	10	5.0	1.7–8.4
Other treatments	75	45.6	36.5–54.6
Traditional treatment	6	3.4	0.5–6.3
No treatment	61	36.5	27.7–45.3
			
*Lenient definition of AS+AQ coverage*^‡^			
AS+AQ	20	14.1	7.6–20.3
Quinine	10	5.0	1.7–8.4
Other treatments	68	41.0	32.1–50.0
Traditional treatment	6	3.4	0.5–6.3
No treatment	61	36.5	27.7–45.3
			
*Treatment coverage with any antimalarial*			
AS+AQ/Quinine	30	19.1	12.1–26.0
Other treatments	68	41.0	32.1–50.0
Traditional treatment	6	3.4	0.5–6.3
No treatment	61	36.5	27.7–45.3
Total	165	100.0	

* Weighted percentage

^† ^Received AS+AQ in a public health centre within 48 hours after onset of fever

^‡ ^Received AS+AQ

### Case management of probable malaria cases

Due to questionnaire misinterpretation, information concerning the place and costs of treatment of probable malaria cases was only collected in 12/18 health catchment areas (99/165 children defined as true positive malaria cases). The most frequented treatment places were public health centres (23/99, 23.3%, 95%CI 12.6–33.9), followed by private clinics (15/99, 14.1%, 95%CI 6.7–21.6). All the other possibilities for case management – i.e. treatment already available at home (5/99, 9.0%, 95%CI 0.0–18.2); treatment bought at markets, private pharmacies or hospitals (15/99, 14.1%, 95%CI 6.7–21.6); traditional treatment (2/99, 0.8%, 95%CI 0.0–2.1) – were mentioned less than 10% in the interviewed households. However, 37 (35.8%, 95%CI 23.4–48.1) did not receive any treatment.

AS+AQ was prescribed in public or private health centres, and was not available in markets or private pharmacies. Among the 23 children who received treatment in a public health centre, 5 (29.4%, 95%CI 1.9–57.0) got AS+AQ and 5 quinine (17.4%, 95%CI 0.0–35.0) as first-line treatment, while 13 (53.2%, 95%CI 25.4–81.0) did not receive any antimalarial treatment.

Altogether, 64.2% (62/99, 95%CI 51.9–76.6) of children received some drug treatment. The median price people had to pay for AS+AQ treatment was 0.5 US$ (IQR: 0.3–1.3 US$), but varied from 0.4 US$ (IQR: 0.2–0.6 US$) in public health centres to 1.3 US$ (IQR: 0.5–1.5 US$) in private health centres. The median price for quinine was 0.6 US$ (IQR: 0.2–1.0 US$). For treatment other than antimalarials (i.e. paracetamol, aspirin, antibiotics, unknown) households paid between 0.3 US$ and 5.0 US$ (Table 3).

**Table 3:** Reported price and place of treatment for probable malaria cases who received any drugs (n = 48/99 with complete information), Makamba province, Burundi, 2004

Treatment	Place of treatment	Median [US$]	IQR [US$]	N
AS+AQ	Public health centres	0.4	0.2–0.6	5
	Private clinics	1.3	0.5–1.5	4
	All sources	0.5	0.3–1.3	9
Quinine	Public health centres	0.7	0.4–1.4	5
	Private pharmacy	0.2		1
	All sources	0.6	0.2–1.0	6
Chloroquine	All sources	0.1		1
Paracetamol/aspirin	All sources	0.3	0.1–0.8	18
Antibiotics	All sources	0.7	0.4–1.9	6
Others	All sources	5.0	5.0–5.0	2
Unknown	All sources	0.7	0.4–2.0	6

## Discussion

This study was the first to evaluate ACT coverage in an African country after its implementation. The results were ambivalent: on the one hand AS+AQ was the most frequently prescribed antimalarial in public health centres and replaced the less effective chloroquine and sulphadoxine-pyrimethamine. On the other hand, treatment coverage was very low. Only one out of 10 probable malaria cases in Makamba province received AS+AQ in a public health centre within two days of fever onset, still far below the goal of the Roll Back Malaria Initiative, namely 60% coverage of childhood fevers with effective antimalarial treatment [4,16,17].

Antimalarial treatment coverage, whichever way is defined, seems poor wherever it has been measured, and study findings are in line with published estimates. Only 7% of childhood fevers were treated promptly with the recommended sulphadoxine-pyrimethamine regimen in Kenya [18], whereas treatment coverage of 33% and 47% were estimated in Tanzania and Gambia, respectively [19,20].

Low utilisation of health centres and apparently inappropriate diagnosis and prescriptions were equally important contributors to low coverage. Only slightly more than half of the probable malaria cases sought formal care. Past reviews in Africa [21-23] have shown unequivocally that home-based treatment is almost always the first choice for patients or caregivers, and that formal or informal health providers are consulted when symptoms are considered unmanageable at home. Apart from Zimbabwe [24] and the Gambia [25], most studies suggest that less than half of febrile children are brought to health centres, and as few as 17% in Burkina Faso [26]. In this study, a worrying proportion of febrile children and probable malaria cases were apparently given no treatment whatsoever, suggesting fundamental financial barriers to health care access, as shown previously in Burundi [27]. Of note is that AS+AQ was given out to patients at a price ten times higher than theoretically fixed. Insufficient payment for personnel in health centres might lead to an inflation of actual point-of-care pricing [28].

Quality of care in public health centres seemed unsatisfactory. These facilities were supposed to have the sole stocks of AS+AQ, to diagnose malaria cases based on a standardized algorithm including parasitological confirmation, and to treat them at fixed and heavily subsidized prices. However, half of the probable malaria cases seen in public health centres did not receive an antimalarial and were instead treated otherwise. These results might indicate that malaria is not always correctly diagnosed in a health centre, and once it is correctly diagnosed prescribing patterns are erratic, as described in other studies performed in Africa [29-31].

In the literature, fever stands often synonymously for malaria [11,12,32-34]. This definition runs the risk of retrospectively overestimating the number of true malaria cases. At least among children, fever or history of fever, irrespective of other symptoms, is highly sensitive, but poorly specific for true malaria [35-37]. Moreover, in a setting where malaria should in accordance to local guidelines only be treated after being diagnosed parasitologically, treatment coverage studies should attempt to identify true malaria cases in the community. In light of this, this study attempted to construct a locally valid symptom-based malaria definition that could be used to identify true malaria cases among children with a history of fever. However, the retrospective definition of malaria had its limitations.

**Figure 2:**
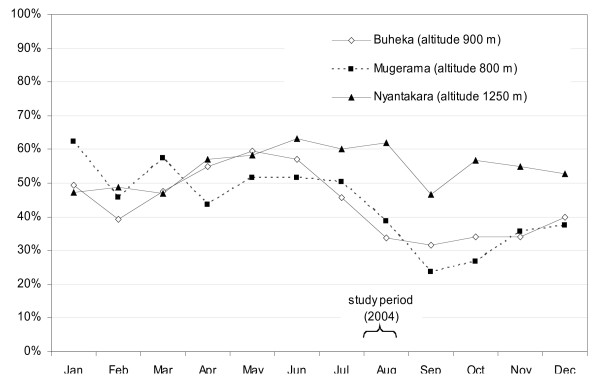
Proportion of under 5 years consultations due to malaria in clinics using rapid diagnostic tests, Makamba province, Burundi, 2003 (source: Medécins Sans Frontières).

Several studies have attempted to establish symptomatic algorithms to diagnose malaria cases presumptively [38-45], but chosen syndrome combinations achieved moderate to low predictive values and varied widely across study sites. No existing method to retrospectively define malaria cases could be found in the literature. However, the PPV of the symptom-based case definition was disappointingly low, and the probability that past malaria cases were indeed true cases was not higher than 60% – probably a relatively small improvement over fever alone as a criterion. PPV is affected by disease prevalence, in this case the proportion of fever cases due to malaria (47.2% in this study). This proportion varies with malaria transmission. Makamba province features very heterogeneous endemicity, ranging from stable, hyper-endemic lowlands to meso-endemic plateau areas to unstable highland malaria. This study was done in July–August, two months into the May–October dry season, i.e. probably still in a high-transmission period, considering the usual two-months delay between rainfall and malaria incidence peaks. While reliable 2004 data are unavailable, monthly trends in the proportion of under 5 years consultations due to malaria in 2003 are shown in Figure 2 for three MSF clinics using a rapid test for systematic case confirmation. The proportion of fever cases due to malaria could also vary based on the incidence of other febrile illnesses.

In light of the less than optimal case definition, coverage estimates must be interpreted with caution. This limitation does not, however, fundamentally alter the main finding, since coverage was obviously low regardless of which denominator was used. Nevertheless, improved methodological approaches to measure antimalarial coverage are sorely needed. An alternative to the method used could have been a qualitative study [46,47], employing in-depth interviews, illness narratives, focus groups and other qualitative techniques to explore local concepts relating to malaria and its treatment, so as to establish a local terminology for malaria and construct a more appropriate questionnaire.

This study's cross-sectional design yields point-in-time estimates that are useful for setting a baseline and monitoring trends in coverage over time. In order to be comparable, further studies would need to be conducted under similar malaria transmission conditions, i.e. during the same season. The disadvantage of this approach is possible seasonal bias (due to the effects of transmission or other factors, such as regular harvesting or food scarcity, on febrile disease morbidity and on access to health care). Ideally, such cross-sectional studies should take place more than once a year.

It should also be noted that the study was carried out in only one of the 16 provinces in Burundi, and thus conclusions cannot be drawn for the entire country.

## Conclusion

With the development of ACT and the change of national malaria protocols in many African countries, a big step has been taken towards effective control of this deadly disease. However, this study demonstrates that this effort may not be sufficient, and illustrates the importance of monitoring coverage and developing better methodologies for doing so. Better access to care is essential to improve treatment coverage; diagnostic and prescribing practices in health centres need to be supervised, and malaria treatment guidelines need to be enforced, including free availability of ACT. Where these conditions are not met, the deployment of new and highly efficacious regimens will probably have a negligible impact on malaria control.

## Competing interests

The author(s) declare that they have no competing interests.

## Authors' contributions

SG participated in the design of the study, conducted the field part and the analysis and wrote the manuscript. SC participated in the study implementation, its analysis, data interpretation, and manuscript revision. KE participated in the study implementation and manuscript revision. AL carried out all microscopic examination of blood samples. CB interpreted the study findings and revised the manuscript. JPG had been involved in the study design and revised the manuscript critically. FC conceived the study, had been involved in drafting the manuscript and revised it critically. All authors read and approved the final manuscript.

## References

[B1] Nosten F, Brasseur P (2002). Combination therapy for malaria: the way forward?. Drugs.

[B2] Whiten NJ, Olliaro PL (1996). Strategies for the prevention of antimalarial drug resistance: rationale for combination chemotherapy for malaria. Parasitol Today.

[B3] Nosten F, van Vugt M, Price R, Luxemburger C, Thway KL, Brockman A, McGready R, ter Kuile F, Looareesuwan S, White NJ (2000). Effects of artesunate-mefloquine combination on incidence of *Plasmodium falciparum *malaria and mefloquine resistance in western Thailand: a prospective study. Lancet.

[B4] Hung Q, Vries PJ, Giao PT, Nam NV, Binh TQ, Chong MT, Quoc NT, Thanh TN, Hung LN, Kager PA (2002). Control of malaria: a successful experience from Viet Nam. Bull World Health Organ.

[B5] Barnes KI, Durrheim DN, Little F, Jackson A, Mehta U, Allen E, Dlamini SS, Tsoka J, Bredenkamp B, Mthembu DJ, White NJ, Sharp BL (2005). Effect of artemether-lumefantrine policy and improved vector control on malaria burden in KwaZulu-Natal, South Africa. PloS Medicine.

[B6] Ministère de la Santé Publique (2003). La prise en charge du paludisme au Burundi: module de formation à l'intention des prestataires des soins.

[B7] (2004). Burundi at a glance.

[B8] Legros D, Dantoine F (2001). Epidémie de paludisme du Burundi, Septembre 2000 – Mai 2001. Epicentre report.

[B9] (2005). World Development Indicators database: Burundi Data Profile.

[B10] Bureau Provincial de l'Etat Civil [Burundi] (2004). Population.

[B11] Amin AA, Marsh V, Noor AM, Ochola SA, Snow RW (2003). The use of formal and informal curative services in the management of paediatric fevers in four districts in Kenya. Trop Med Int Health.

[B12] Monasch R, Reinisch A, Steketee RW, Korenromp EL, Alnwick D, Bergevin Y (2004). Child coverage with mosquito nets and malaria treatment from population-based surveys in african countries: a baseline for monitoring progress in roll back malaria. Am J Trop Med Hyg.

[B13] Lubanga RG, Norman S, Ewbank D, Karamagi C (1997). Maternal diagnosis and treatment of children's fever in an endemic malaria zone of Uganda: implications for the malaria control programme. Acta Trop.

[B14] Williams HA, Jones C, Alilio M, Zimicki S, Azevedo I, Nyamongo I, Sommerfeld J, Meek S, Diop S, Bloland PB, Greenwood B (2002). The contribution of social science research to malaria prevention and control. Bull World Health Organ.

[B15] Myatt M (2001). SampleLQ-a sample size calculator for community-based triage surveys using lot quality assurance sampling.

[B16] World Health Organisation (2005). African Summit on Roll Back Malaria: Abuja Summary Report.

[B17] World Health Organisation (2003). Protocols and methods for malaria situation analysis.

[B18] Guyatt HL, Snow RW (2004). The management of fevers in Kenyan children and adults in an area of seasonal malaria transmission. Trans R Soc Trop Med Hyg.

[B19] Von Seidlein L, Clarke S, Alexander N, Manneh F, Doherty T, Pinder M, Walraven G, Greenwood B (2002). Treatment uptake by individuals infected with *Plasmodium falciparum *in rural Gambia, West Africa. Bull World Health Organ.

[B20] Alilio MS, Kitua A, Njunwa K, Medina M, Ronn AM, Mhina J, Msuya F, Mahundi J, Depinay JM, Whyte S, Krasnik A, Bygbjerg IC (2004). Malaria control at the district level in Africa: the case of the Muheza district in northeastern Tanzania. Am J Trop Med Hyg.

[B21] Williams HA, Jones CO (2004). A critical review of behavioral issues related to malaria control in sub-Saharan Africa: what contributions have social scientists made?. Soc Sci Med.

[B22] McCombie SC (2002). Self-treatment for malaria: the evidence and methodological issues. Health Policy Plan.

[B23] McCombie SC (1996). Treatment seeking for malaria: a review of recent research. Soc Sci Med.

[B24] Tsuyuoka R, Wagatsuma Y, Makunike B (2001). The knowledge and practice on malaria among community members in Zimbabwe. Cent Afr J Med.

[B25] Clarke SE, Rowley J, Bogh C, Walraven GE, Lindsay SW (2003). Home treatment of 'malaria' in children in rural Gambia is uncommon. Trop Med Int Health.

[B26] Muller O, Traore C, Becher H, Kouyate B (2003). Malaria morbidity, treatment-seeking behaviour, and mortality in a cohort of young children in rural Burkina Faso. Trop Med Int Health.

[B27] (2005). Burundi-Vulnerable population deprived of healthcare.

[B28] Rowe AK, Onikpo F, Lama M, Cokou F, Deming MS (2001). Management of childhood illness at health facilities in Benin: problems and their causes. Am J Public Health.

[B29] Amexo M, Tolhurst R, Barnish G, Bates I (2004). Malaria misdiagnosis: effects on the poor and vulnerable. Lancet.

[B30] Nshakira N, Kristensen M, Ssali F, Whyte SR (2002). Appropriate treatment of malaria? Use of antimalarial drugs for children's fevers in district medical units, drug shops and homes in eastern Uganda. Trop Med Int Health.

[B31] Zurovac D, Ndhlovu M, Rowe AK, Hamer DH, Thea DM, Snow RW (2005). Treatment of paediatric malaria during a period of drug transition to artemether-lumefantrine in Zambia: cross sectional study. BMJ.

[B32] Deming MS, Gayibor A, Murphy K, Jones TS, Karsa T (1989). Home treatment of febrile children with antimalarial drugs in Togo. Bull World Health Organ.

[B33] Molyneux CS, Mung'Ala-Odera V, Harpham T, Snow RW (1999). Maternal responses to childhood fevers: a comparison of rural and urban residents in coastal Kenya. Trop Med Int Health.

[B34] Verhoef H, Hodgins E, Eggelte TA, Carter JY, Lema O, West CE, Kok FJ (1999). Anti-malarial drug use among preschool children in an area of seasonal malaria transmission in Kenya. Am J Trop Med Hyg.

[B35] Bell D, Go R, Miguel C, Walker J, Cacal L, Saul A (2001). Diagnosis of malaria in a remote area of the Philippines: comparison of techniques and their acceptance by health workers and the community. Bull World Health Organ.

[B36] Tarimo DS, Minjas JN, Bygbjerg IC (2001). Malaria diagnosis and treatment under the strategy of the integrated management of childhood illness (IMCI): relevance of laboratory support from the rapid immunochromatographic tests of ICT Malaria P.f/P.v and OptiMal. Ann Trop Med Parasitol.

[B37] Perkins BA, Zucker JR, Otieno J, Jafari HS, Paxton L (1997). Evaluation of an algorithm for integrated management of childhood illness in an area of Kenya with high malaria transmission. Bull World Health Organ.

[B38] Muhe L, Oljira B, Degefu H, Enquesellassie F, Weber MW (1999). Clinical algorithm for malaria during low and high transmission seasons. Arch Dis Child.

[B39] Gomes M, Espino FE, Abaquin J, Realon C, Salazar NP (1994). Symptomatic identification of malaria in the home and in the primary health care clinic. Bull World Health Organ.

[B40] Chandramohan D, Carneiro I, Kavishwar A, Brugha R, Desai V, Greenwood B (2001). A clinical algorithm for the diagnosis of malaria: results of an evaluation in an area of low endemicity. Trop Med Int Health.

[B41] Chandramohan D, Jaffar S, Greenwood B (2002). Use of clinical algorithms for diagnosing malaria. Trop Med Int Health.

[B42] Genton B, Smith T, Baea K, Narara A, Al Yaman F, Beck HP, Hii J, Alpers M (1994). Malaria: how useful are clinical criteria for improving the diagnosis in a highly endemic area?. Trans R Soc Trop Med Hyg.

[B43] Bojang KA, Obaro S, Morison LA, Greenwood BM (2000). A prospective evaluation of a clinical algorithm for the diagnosis of malaria in Gambian children. Trop Med Int Health.

[B44] Olaleye BO, Williams LA, D'Alessandro U, Weber MM, Mulholland K, Okorie C, Langerock P, Bennett S, Greenwood BM (1998). Clinical predictors of malaria in Gambian children with fever or a history of fever. Trans R Soc Trop Med Hyg.

[B45] Redd SC, Kazembe PN, Luby SP, Nwanyanwu O, Hightower AW, Ziba C, Wirima JJ, Chitsulo L, Franco C, Olivar M (1996). Clinical algorithm for treatment of *Plasmodium falciparum *malaria in children. Lancet.

[B46] Ahorlu CK, Dunyo SK, Afari EA, Koram KA, Nkrumah FK (1997). Malaria-related beliefs and behaviour in southern Ghana: implications for treatment, prevention and control. Trop Med Int Health.

[B47] Winch PJ, Makemba AM, Kamazima SR, Lurie M, Lwihula GK, Premji Z, Minjas JN, Shiff CJ (1996). Local terminology for febrile illnesses in Bagamoyo District, Tanzania and its impact on the design of a community-based malaria control programme. Soc Sci Med.

